# *p*HUSH: a single vector system for conditional gene expression

**DOI:** 10.1186/1472-6750-7-61

**Published:** 2007-09-26

**Authors:** Daniel C Gray, Klaus P Hoeflich, Li Peng, Zhenyu Gu, Alvin Gogineni, Lesley J Murray, Mike Eby, Noelyn Kljavin, Somasekar Seshagiri, Mary J Cole, David P Davis

**Affiliations:** 1 Department of Molecular Biology, Genentech Inc, South San Francisco, California, USA; 2 Department of Translational Oncology Genentech Inc, South San Francisco, California, USA; 3 Department of Biomedical Imaging Group, Genentech Inc, South San Francisco, California, USA; 4 Department of Internal Medicine, UC Davis Cancer Center, University of California, Davis, California, USA; 5 Antibody Discovery and Protein Engineering Department, MedImmune Inc, Gaithersburg, Maryland, USA

## Abstract

**Background:**

Conditional expression vectors have become a valuable research tool to avoid artefacts that may result from traditional gene expression studies. However, most systems require multiple plasmids that must be independently engineered into the target system, resulting in experimental delay and an increased potential for selection of a cell subpopulation that differs significantly from the parental line. We have therefore developed pHUSH, an inducible expression system that allows regulated expression of shRNA, miRNA or cDNA cassettes on a single viral vector.

**Results:**

Both Pol II and Pol III promoters have been successfully combined with a second expression cassette containing a codon-optimized tetracycline repressor and selectable marker. We provide examples of how pHUSH has been successfully employed to study the function of target genes in a number of cell types within *in vitro *and *in vivo *assays, including conditional gene knockdown in a murine model of brain cancer.

**Conclusion:**

We have successfully developed and employed a single vector system that enables Doxycycline regulated RNAi or transgene expression in a variety of in vitro and in vivo model systems. These studies demonstrate the broad application potential of pHUSH for conditional genetic engineering in mammalian cells.

## Background

The development of RNA interference (RNAi) as a tool for reverse genetic studies in mammalian systems has rapidly matured. After the seminal observation that 21 nucleotide, chemically-synthesized RNA duplexes (referred to as short-interfering RNA or siRNA) are capable of targeted gene silencing in mammalian cells [1], RNAi has quickly become a standard technique for functional genetic analysis. A significant advancement of this technique was the development of short-hairpin RNA (shRNA) expression technology [2,3]. This strategy exploits the defined transcriptional start and termination signals of RNA polymerase III (Pol III) promoters to produce a short, inverted transcript. These stem-loop RNA transcripts are then processed within the cell into functional siRNAs and thereby provide a means for the stable suppression of target genes. To this end, multiple groups have reported success in long-term silencing of target genes in engineered cell lines and mice [4,5].

Nevertheless, several limitations to the current approach remain. The primary limitation to vector-based shRNA is posed by constitutive hairpin expression. If the shRNA is directed against a gene essential to cell growth and survival, the probability of obtaining a stable line is low, and in those cell lines that survive, other factors may compensate for shRNA-induced gene knockdown. In both cases, the relevant phenotype may be obscured [6]. Several groups have employed inducible shRNA systems to address these limitations. One class of inducible systems co-express the tetracycline repressor (TetR) with a modified Pol III promoter containing one or more TetR operons flanking the TATA-box such that transcription is blocked when the TetR is bound to the promoter. The expression of shRNA within this scenario occurs in the presence of tetracycline or related analogs [7-14].

Wiznerowicz and Trono introduced a variation to the above design by fusing the TetR to KRAB, a transcriptional repression domain from Kox1 [15]. This fusion silences any promoter within 3 kb of the TetR operon. Therefore, shRNA transcription from the Pol III promoter containing a TetR operon may also be monitored by the ability of the TetR-KRAB fusion to repress the expression of a fluorescent reporter gene driven by an additional RNA polymerase II (Pol II) promoter within the same vector [15]. In a different approach, several labs have developed Cre recombinase-based systems in which the shRNA promoter is modified to contain a 'floxed' cassette or spacer such that hairpin expression is activated only upon Cre-mediated excision [16,17]. In addition to standard or 'first generation' shRNA design, it has recently been demonstrated that a primary micro-RNA (miRNA) scaffold can efficiently deliver a desired shRNA [18,19]. These 'second-generation' miRNA mimetics can be expressed from Pol II promoters, allowing the use of established conditional expression vectors [20,21].

Each of the above classes of regulated shRNA vectors has specific advantages – from the temporal and reversible control of the tetracycline-regulated systems to tissue-specific silencing of Cre-lox vectors in mice. However, a considerable limitation shared by the reported inducible shRNA systems is that each system relies on a two-plasmid approach in which the shRNA expression cassette is separated from the regulatory module. To address this issue, we and others have previously reported the successful integration of a shRNA/miRNA expression cassette with a tetracycline dependent repressor or transactivator on a single vector [22-27]. Here we describe the development of pHUSH (H1 or U6 short hairpin), as a Gateway^®^-compatible, conditional shRNA system in which TetR expression and operon usage have been optimized on a single vector. Importantly, we compare a number of different shRNA and miRNA configurations in transient transfection assays and show that the pHUSH backbone is effective at providing regulated expression from both pol II and pol III promoters, thereby enabling regulated shRNA, miRNA and protein expression. The versatility of pHUSH is demonstrated here using a series of *in vitro *and *in vivo *reverse genetic experiments that were designed to validate the therapeutic relevance of both novel and characterized oncology targets.

## Results

### Analysis of shRNA design

**Figure 1:**
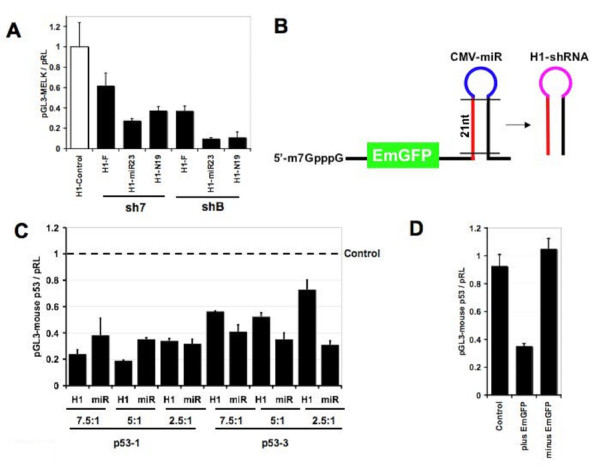
Luciferase-based transient co-transfection experiments to compare shRNA and miRNA design schemes. Knockdown studies were performed in duplicate as co-transfections of shRNA:pGL3-gene:pRL at the indicated ratio. Firefly luciferase values are divided by renilla values to control for transfection efficiency, and all knockdown data are normalized to control hairpins. (A) Comparison of published modifications to standard H1-shRNA design (F = frayed, miR23 = loop derived from miR-23, and N19 = standard pSuper shRNA design) directed against the pGL3-MELK reporter. Cells were transfected with a 10:1 ratio of the indicated shRNA to pGL3-Melk reporter. (B) CMV-miR to H1-shRNA conversion scheme. The active 'siRNA' was identified within the pol II-miRNA and converted to a standard H1-shRNA hairpin (black bars = Drosha and Dicer processing; red = guide strand, blue = miR-155 loop, pink = pSuper loop). (C) Comparison of H1-shRNA and CMV-miR vectors corresponding to murine p53 sequences transfected at 7.5:1, 5:1 and 2.5:1 (shRNA:pGL3-mu-p53). (D) The removal of the EmGFP leader renders miR-p53-1 non-functional at a co-transfection ratio of 10:1.

As part of our effort to engineer a conditional shRNA expression system, we compared hairpin stem and loop parameters to maximize knockdown efficiency. To generate a robust, quantifiable assay, we created a transcript fusion between luciferase and Melk, a Ser/Thr kinase that we have previously described [25]. The entire Melk cDNA was cloned downstream of the pGL3-Luc stop codon, and the resulting pGL3-Melk construct was used as a reporter for shRNA efficacy in transient co-transfection experiments. We first compared several published strategies for shRNA design based on two target sequences: sh7 (nt 1686–1704) and shB (nt 1917–1935). For both target sequences, the following series of shRNAs were created for comparison: N19 – a simple stem-loop design [2], F – a 'frayed' shRNA design employing artificial asymmetry [28], and mi23 – a shRNA utilizing the miR23 loop reported to increase cytoplasmic expression [29,30]. The sequence of each hairpin is presented in additional file 1. To compare the efficacy of each design, the resulting shRNA expression constructs were co-transfected with the pGL3-Melk reporter at several molar ratios and normalized to control hairpin H1-LacZ. As shown in Figure 1A at a 10:1 ratio of shRNA to pGL3-MELK, luciferase expression was decreased for both shRNA sequences, regardless of the hairpin design. The standard N19 design and mi23-loop shRNAs were highly effective, yielding 65% (H1-sh7-N19), 75% (H1-sh7-mi23), 90% (H1-shB-N19), and 95% (H1-shB-mi23) knockdown respectively. While we noticed a modest increase in knockdown efficiency by the addition of the miR23 loop at a 10:1 ratio of shRNA to pGL3-MELK, there was no significant increase in efficacy with the miR23 loop at lower molar ratios of shRNA to pGL3-MELK (data not shown) as had been previously reported [30]. For both sh7 and shB, the contribution of artificial thermodynamic symmetry (shRNA-F) by the introduction of 'frayed' ends at the 3' end of the sense strand was detrimental to hairpin efficacy, suggesting that destabilization of the shRNA stem-loop in this context did not lead to increased RISC loading of the antisense strand. As there did not appear to be a reproducible improvement with modified shRNAs (F or miR23), we have utilized a N19 shRNA design for the remainder of this study.

Over the course of our study, several labs reported robust knockdown with miRNA-based vectors [18-21]. Using a luciferase transcript-fusion construct with murine p53 sequences cloned downstream of the pGL3-Luc stop codon, we compared the 'first generation' H1-shRNA (N19) to a 'second-generation' CMV-miRNA-155 vector [19]. Unlike the N19 shRNA design, the miRNA mimetic can be expressed as standard pol II transcripts, which are then processed by the nuclear Drosha, and cytoplasmic Dicer endonucleases to form a 21–22 nt active siRNA [31]. To enable this comparison, we generated a vector in which a modified CMV promoter [32] was used to express Emerald GFP (EmGFP) and the miRNA mimetic on the same transcript (CMV/TO-EmGFP-miRNA). To compare the efficacy of a shRNA versus miRNA design, two effective miRNAs directed against p53 were converted to H1-shRNAs by identifying the 21 nt active siRNA within the miRNA scaffold and then converting these sequences into conventional shRNA design according to the scheme presented in Figure 1B. The sequence and structure of these hairpins is presented in additional file 2. Although the performance of the CMV/TO-miR and H1-shRNA hairpins using the p53-1 sequence were similar, we observed increased knockdown by CMV/TO-miR relative to the corresponding H1-shRNA sequence for the p53-3 sequence (Figure 1C). At the lowest ratios of miR or shRNA to reporter (2.5:1 or 5:1), the CMVTO-miR resulted in ~65–70% knockdown of the luciferase reporter as compared to only a ~30–50% knockdown with the H1-shRNA (Figure 1C). Removing the EmGFP leader from CMV-miR-p53-1 destroyed the knockdown efficiency of the original construct (Figure 1D), confirming the requirement of a minimal "spacer" sequence between the transcriptional start site and the miRNA sequence [21]. To summarize, we have directly compared "first-generation" pol III-shRNA vectors to "second-generation" pol II vectors using the same Dicer-product siRNAs. We have found that both vector formats are functional. However, based on our observed increase in knockdown efficiency with CMV/TO-miR for the p53-3 sequence as compared to the corresponding H1-shRNA version, there may be a slight advantage with the miRNA-like systems for certain sequences under suboptimal expression conditions.

### pHUSH vector design and optimization

**Figure 2:**
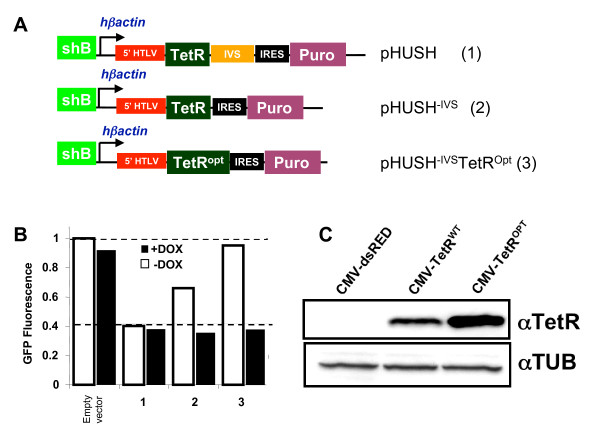
pHUSH vector design and optimization (A) Vector diagrams of the pHUSH vector series: (1) original pHUSH backbone (2) pHUSH^-IVS ^in which the synthetic intron sequence between the TetR ORF and the IRES is removed, and (3) pHUSH^-IVS^TetR^Opt ^with a codon-optimzed TetR (B) Increased TetR expression ensures regulation of GFP-Melk fusion protein in the absence of doxycycline. HEK 293 cells expressing a GFP-MELK fusion were transfected with the pHUSH vector series described above all containing the shB Melk targeting shRNA. After selection with 3 μg/ml puromycin, the resulting stable pools were cultured in the absence (open bars) or presence (filled bars) of 1 μg/ml doxycycline for five days and GFP-Melk expression analyzed by FACS. Data is normalized to the mean fluorescence intensity (10,000 acquired events) for cells containing a pHUSH empty vector control. A representative experiment is shown. (C) Codon optimization of TetR open reading frame (ORF) increases translation. Both the original (WT) and codon-optimized (OPT) TetR ORFs were transiently expressed in 293T cells. Forty-eight hours post-transfection, cell lysates were prepared and Western blotted with an anti-TetR antibody and an anti-tubulin antibody.

pHUSH was constructed as described in the Methods using the pIRES-Puro2 as the backbone vector. Briefly, the human-β-actin promoter driving the TetR was subcloned into pIRES-Puro2 to replace the original CMV promoter. A modified H1 promoter containing a single TetR operon with a Melk-targeting shRNA cassette was inserted upstream to the human-β-actin promoter. Using this plasmid as the base vector, we generated two additional versions for analysis: pHUSH^-IVS ^in which a synthetic intron sequence between the TetR ORF and the IRES was removed, and pHUSH^-IVS^TetR^Opt^, in which the original TetR sequence within pHUSH^-IVS ^was replaced by a TetR expression cassette that was codon-optimized for expression in mammalian cells (Figure 2A). To assess the effect of these modifications on regulated gene silencing, these constructs were transfected into HEK293 cells that were stably expressing an EGFP-Melk fusion protein. After selection with puromycin, cells were cultured for five days in the presence or absence of Doxycycline (Dox), and the level of EGFP-MELK fluorescence was scored by FACS analysis. The effectiveness of each construct to repress shRNA expression was assessed by comparing the level of EGFP-Melk in the absence of Dox to cells containing a pHUSH construct without a hairpin (empty vector). As shown in Figure 2B, all three vectors generated the same level of knockdown in the presence of Dox (filled bars). However, we found that in the absence of Dox (open bars), only the pHUSH^-IVS^TetR^opt ^pool exhibited full repression of shRNA expression (column 3, Figure 2B). In contrast, partial repression (70% of maximal EGFP-Melk) was obtained with pHUSH^-IVS ^while cells containing the base pHUSH vector displayed a complete lack of shRNA repression, as demonstrated by similar levels of EGFP-Melk in the presence or absence of Dox (Figure 2B – columns 2 and 1, respectively). Because of the reported strong splicing donor in the 5' HTLV UTR [33], the removal of the IVS most likely prevented a splicing event between the UTR and the splice acceptor within IVS resulting in the excision of the TetR. The above data suggests optimal Dox-dependent regulation of shRNA expression requires maximal TetR protein levels. To test this hypothesis, we compared TetR expression between the original TetR construct (derived from pcDNA6/TR) and the codon-optimized variant utilized for pHUSH^-IVS^TetR^Opt^. As shown in Figure 2C, there is a two-to-three fold increase in TetR protein levels with the codon-optimized TetR (CMV-TetR^opt^) as compared to the original construct (CMV-TetR^wt^). Therefore, we conclude that both TetR codon optimization and IVS removal are required for a regulated gene silencing system.

**Figure 3:**
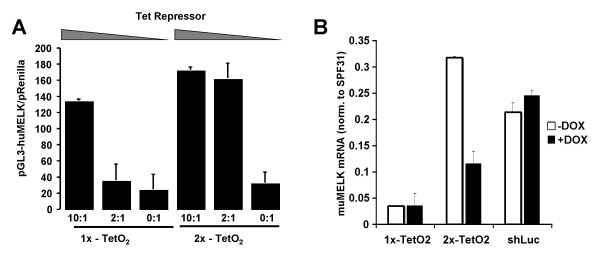
Multiple TetO_2 _operons within the Pol III promoter enhances regulated knock-down. (A) 293T cells were transfected with 15 ng of pGL3-huMelk in the presence of decreasing molar ratios of TetR and the appropriate H1-shRNA vectors using the indicated molar ratio of TetR:H1-shRNA vector. The maximal level of gene knockdown that can be observed with either promoter is represented by cells transfected with the appropriate H1-shRNA vector and no TetR (0:1). Both H1-shRNA constructs expressed the shB Melk targeting shRNA. Luciferase expression was measured as described in the Methods 48 hours post transfection. (B) Dox regulated Melk knockdown is maintained in differentiated ES cells with the 2 × TetO_2 _promoter configuration. Embryoid bodies were generated as described in the Methods from stable pHUSH ES cell clones with shRNA targeting MELK or Luciferase. shMELK ES cell lines were generated with either one TetO_2 _(1×-TetO2, colony A6) or two TetO_2 _(2×-TetO2, colony 3C11) operons (see also additional file 3). ES cells lines were differentiated into embryoid bodies in the presence or absence of 1 ug/ml doxycycline and the level of Melk expression determined by qRT-PCR and normalized to the housekeeping gene SPF31 (RefSeq NM_014280).

Because of the observed sensitivity to modest changes in TetR levels, we next focused on further modifying the H1 promoter to ensure complete shRNA repression under limiting TetR levels. Several groups have shown that increased regulation can be achieved by inserting multiple TetR operons throughout the Pol III promoter [9,32,34]. To determine whether we could observe a similar enhancement of regulation, we modified the original pShuttle-H1-shRNA plasmid containing a single TetO_2 _(1×-TetO_2_) with an additional TetO_2 _operon upstream of the TATA box, forming a 2×-TetO_2_-H1 promoter. Using pGL3-Melk as a reporter, the 1×- and 2×-TetO_2_-H1 promoters were co-transfected with increasing amounts of a CMV-TetR^Opt ^expression plasmid in the absence of Dox. For both shRNA promoter variants, maximal repression was achieved when the TetR expression construct was supplied in a 10:1 molar excess over the H1-shRNA (Figure 3A, 10:1) while >80% knockdown was achieved in the absence of TetR (Figure 3A, 0:1). When the TetR expression plasmid was limited to a 2:1 molar ratio relative to the H1-shRNA plasmid, nearly maximal shRNA expression was observed with the 1×-TetO_2 _construct. In contrast, shRNA repression was maintained with the 2×-TetO_2 _construct (Figure 3A). These results suggest that the use of two TetR operons enhance the ability to regulate shRNA expression when TetR expression is limiting.

To explore the robustness of this system as a stably-integrated construct, we then compared the 1×-TetO_2 _and 2×-TetO_2 _versions of the pHUSH vector to enable regulated silencing of Melk within mouse embryonic stem (ES) cells. Murine ES cells were electroporated with pHUSH^-IVS^TetR^opt ^or pHUSH^-IVS^TetR^opt^2 × TetO_2_, containing a Melk-directed shRNA and then selected in puromycin. Multiple clones for both versions of pHUSH were identified exhibiting robust, regulated MELK knockdown (>70–90%) after 48 h culture in the presence of Dox (additional file 3). To determine whether regulated shRNA expression could be maintained upon differentiation, select ES cell clones (A6 and 3C11) were allowed to differentiate into embryoid bodies (EBs) by standard methods. EB formation was induced and cultured in the presence or absence of 1 ug/ml Dox for 14 days, and endogenous Melk RNA levels were quantified by qRT-PCR. As shown in Figure 3B, near maximal silencing of Melk expression was observed with EBs derived from pHUSH^-IVS^TetR^opt ^(1×-TetO2, clone A6) without DOX, indicating no functional repression of shRNA expression. In contrast, EBs derived from pHUSH^-IVS^TetR^opt^2×-TetO_2 _(2×-TetO2, clone 3C11) maintained a similar level of Dox-dependent regulation as had been observed with the parental, non-differentiated ES cells (additional file 3). Consistent with these results, we observed a 3-fold decrease in TetR expression upon differentiation into EBs (data not shown). Although the overall level of Melk knockdown is reduced within the differentiated EB derived from the 2×-TetO2 promoter relative to the 1×-TetO2 version (Figure 3B), this difference is likely due to clonal variations during ES cell selection rather than an inherent difference in transcriptional strength of the modified H1 promoter. This is supported by the fact that the knockdown efficiency between the 1× and 2× TetO2 constructs is the same when expressed in the absence of TetR (Figure 3A). In summary, the presence of multiple TetR operons within the H1 promoter does not significantly effect maximal shRNA expression while promoting Dox regulated gene silencing even under limiting TetR expression levels.

### pHUSH Retroviral Vectors

**Figure 4:**
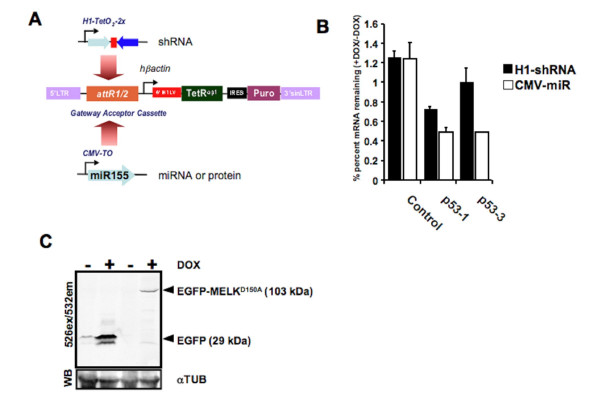
Retroviral delivery of the pHUSH system allows the inducible expression of shRNA, miRNA or proteins. (A) Vector diagram of the pHUSH retroviral system. Inducible pol II or pol III expression cassettes are introduced by Gateway^® ^recombination. (B) Improved p53 knockdown by CMV-miRNA in comparison to H1-shRNA vectors at low MOI. H1-shRNA and CMV-miRNA vectors with hairpins designed against murine p53 as described in additional file 2 were cloned into the pHUSH retroviral backbone. 3T3 cells were infected at an MOI = 0.2, selected in 1.5 ug/mL puromycin, and cultured ± 1 ug/mL Dox, Knockdown of p53 was determined by qRT-PCR at Day 4 post-Dox and was normalized to p53 levels in the absence of Dox. (C) Inducible expression of proteins by the pHUSH retroviral system. Inducible EGFP and EGFP-MELK-D150A expression cassettes were cloned into the pHUSH retroviral backbone. HCT116 cells were infected with the appropriate viral construct, selected in 5 ug/mL puromycin, and treated ± 1 ug/mL Dox for 72 h. Equal total protein was fractionated by non-reducing SDS-PAGE and imaged by the Typhoon fluorescent imager at the indicated excitation and emission spectra (WB = western blot). Equal loading was confirmed by western blotting with an anti-tubulin antibody.

To enhance the stable delivery into cultured cells, the pHUSH^-IVS^TetR^opt^2×-TetO_2 _described above was subcloned into the pQCXIP retroviral backbone to form pHUSH-Retro. The resulting viral pHUSH plasmid was converted to a Gateway^®^-compatible vector to enable efficient transfer of shRNA cassettes between vectors. To compare the ability to conditionally express shRNA or miRNA using the retroviral backbone, the H1–2 × Tet02 and CMV-TO vectors containing p53 shRNA or miRNA, respectively, were introduced into the pHUSH-Retro-Puro backbone by Gateway^® ^recombination (Figure 4A). Viral supernatants of pHUSH-Retro were prepared as described in the Methods and used to infect 3T3 cells at a multiplicity of infection of about 0.2. Cells were selected in 1.5 ug/mL puromycin and then cultured in the presence or absence of 1 ug/mL Dox for four days. Interestingly, both CMV-miR-p53 vectors were more effective than the corresponding H1-shRNA vectors (Figure 4B). Consistent with the transient transfection results (Figure 1C) the p53-1 sequence was more effective than p53-3. Moreover, these results indicate that inducible and stable target gene at a single copy can be achieved by employing 'second generation' CMV-miR hairpin design and are in agreement with previous reports [20,21]. It is important to note, however, that robust silencing using the H1-shRNA vectors can be achieved presumably as the result of multiple integrations per cell (Figure 3 and [25,26]). A simple strategy we have found for maximizing the level of silencing when using the H1-shRNA pHUSH vectors is to escalate the selection pressure downstream of the TetR (additional file 4)

Finally, we also provide evidence that regulated protein expression can be achieved using the pHUSH backbone. For this purpose, the EmGFP-miR sequence of CMV-TO-EmGFP-miRNA was replaced by EGFP or EGFP-MELK-D150A, a previously described kinase-dead MELK variant [25] fused to the C-terminus of EGFP. pHUSH protein vectors were completed by Gateway^® ^cloning, retroviral stocks were generated by standard methods, and HCT116 cells were serially infected and selected at 5 ug/mL puromycin for two weeks. The resulting stable cell lines were treated ± 1 ug/mL Dox for 72 h. EGFP or EGFP-MELK-D150A expression was analyzed by direct fluorescent imaging of a non-reducing polyacrylamide gel (Figure 4C), and western blotted for tubulin to confirm equal loading. The results indicate that inducible expression of the proteins can also be achieved with this vector configuration and has in fact been successfully employed to conditionally express a temperature-sensitive mutant of the GTPase dynamin-1 [35]. We conclude that the retroviral pHUSH vector is capable of inducible pol III-shRNA expression, pol II-miRNA expression, and pol II-protein expression. At present, we have generated destination pHUSH backbones with the selectable markers as summarized in additional file 6.

#### *In Vivo *shRNA Knockdown by pHUSH

**Figure 5:**
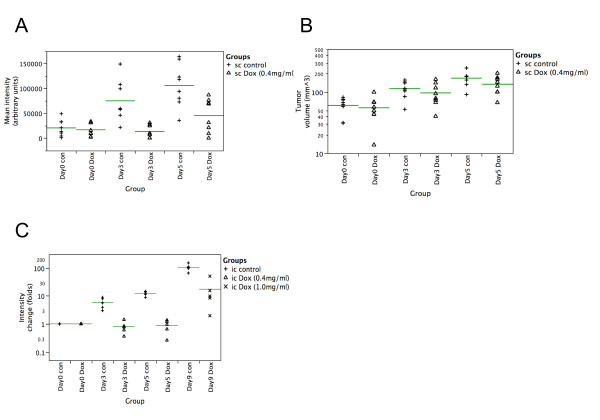
Effective in vivo silencing of luciferase in subcutaneous and intracranial tumor models. (A) Mean intensity luciferase expression indicates Dox induced luciferase knockdown in subcutaneous tumor xenograft at day 3 and 5 post-injection. Eight animals are represented for each treatment condition. (B) Dox treatment or luciferase knockdown does not reduce tumor growth of SVT2 cells in SCID mice. (C) Mean intensity luciferase expression demonstrates Dox-induced luciferase knockdown relative to controls at days 3 (9.5 fold), 5 (13.8 fold) and 9 (11 fold) post-injection. The luminescence values for each time point have been normalized to the respective intensity value at day 0. Five animals are represented for each treatment condition. Representative BLI images are shown in additional file 7.

To demonstrate the utility of pHUSH as a technology by which to interrogate the role of a target gene within relevant tumor models *in vivo*, we constructed a retroviral pHUSH vector with a luciferase-directed shRNA using the H1-TetO2 promoter. Retroviral stocks were prepared as before, and luciferase-expressing SVT2 cells were transduced and selected in 2 ug/mL puromycin. We identified multiple clones that exhibited high luciferase expression without Dox and significant knockdown of luciferase expression *in vitro *(data not shown). A single clone (SVT2 shLuc.pGL3-B3) expressing on average ~200 relative light units per ug of total protein in the absence of Dox and displaying ~60% Dox induced knockdown was selected for in vivo modeling experiments. To this end, immunodefecient *nu/nu *mice were injected subcutaneously with 1 × 10^6 ^cells of SVT-shLuc.pGL3-B3. After tumors had reached 250 mm^3^, the animals were separated into the appropriate Dox treatment group. Tumor growth and luciferase expression were monitored as described in Methods. As shown in Figure 5a and 5b, control animals treated with sucrose water rapidly formed tumors with high luciferase expression, while those dosed with 400 μg/ml Dox in the drinking water maintained rapid tumor growth with low luciferase expression. These studies indicate that functional, Dox regulated shRNA expression can be achieved *in vivo *with no discernable indirect effects on tumor growth or animal health.

We then proceeded to test if inducible, shRNA-mediated knockdown of reporter gene expression in an orthotopic brain tumor model could be achieved. Unlike conventional subcutaneous xenograft models that can be monitored by caliper measurement, accurate in vivo quantitation of intracranial tumor growth necessitates the use of non-invasive imaging techniques. Both MRI and bioluminescence imaging have been previously established as particularly well suited to the monitoring of glioma progression *in vivo *[36]. Since Dox and luciferin have been previously reported to freely cross the brain-blood barrier, we decided to test whether combining shRNA knockdown and biolumenscent imaging may be a method to expedite target validation studies in these more complex tumor models. To this end, SVT2-shLuc.pGL3-3 cells were injected intracranially and tumor growth and luciferase expression was monitored as described in Methods at days 3, 5, and 9 post Dox treatment. We observed Dox mediated knockdown at a day 3 (9.5-fold), day 5 (13.8-fold) and at day 9 (11-fold) (Figure 5c). Tumor size at day 9 as quantitated by luciferase expression strongly correlated with tumor volume as measured ex-vivo by ultrasound (data not shown). Representative images for both subcutaneous and orthotopic experiments are present in additional file 7. These data validate the effectiveness of the pHUSH inducible shRNA system in an orthotopic mouse brain tumor model.

**Figure 6:**
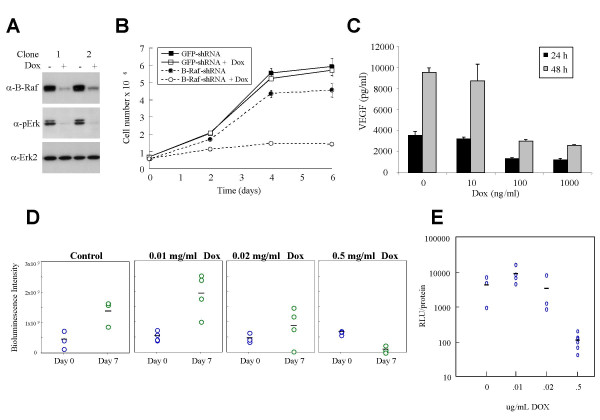
Robust and titratable depletion of oncogenic B-Raf in melanoma tumor lines elicits the expected phenotypes *in vitro *and *in vivo*. (A) Knockdown of BRAF at Day 2 upon Dox addition as determined by western blotting in A375 cells. BRAF knockdown results in a significant reduction in phospho-ERK levels. (B) Proliferation assay reveals a growth stasis phenotype upon BRAF knockown in comparison to a shEGFP control in A375 cells. (C) Dose dependent decrease in VEGF secretion correlates with BRAF knockdown 24 and 48 h post Dox addition. (D) *In vivo *BRAF knockdown detected by non-invasive bioluminescence imaging. A previously characterized LOX-IMV1 clone containing pHUSH-Braf-shRNA [26] was re-engineered to express a Luc-BRAF transcipt fusion. After initiating a subcutaneous tumor model in Scid-beige mice, the expression level of a Braf targeting shRNA was indirectly monitored by bioluminescence imaging at 0, 0.01 mg/mL, 0.02 mg/mL, and 0.5 mg/mL Dox. (E) Day 25 tumors were harvested and equal total protein was assayed for luciferase enzyme activity *in vitro*.

We next sought to combine knockdown of a relevant oncogene with bioluminescence imaging (BLI) within tumor models *in vivo*. We have previously generated and characterized retroviral pHUSH vectors with multiple B-raf-directed shRNAs. B-Raf, a serine/threonine kinase, is mutated in 70% of melanomas [37] and has been proposed as a candidate small molecule therapeutic target [38]. We have previously provided data in support of this proposal by demonstrating the requirement of this kinase within several tumor growth models [26] by employing pHUSH to knockdown the expression of B-Raf in human melanoma cell lines. We have since continued to deplete oncogenic B-Raf in multiple settings and now provide additional data to further demonstrate how this technology may be applied. As shown in Figure 6A, we were successful at generating and identifying clones with Dox-dependent silencing of B-Raf in A375 cells, a human tumor cell line derived from a malignant cutaneous melanoma. We also observed an inhibition of B-Raf signaling upon silencing, demonstrated by a decrease in phospho-Erk1/2 levels. As expected, the consequence of B-raf knockdown and MAPK pathway modulation is evident by a decrease in proliferation under low serum, as well as a Dox-dose dependent decrease in VEGF secretion *in vitro *(Figures 6B and 6C).

To establish a method to non-invasively monitor BRAF silencing within the context of a tumor model *in vivo*, a LOX-IMV1 melanoma line with the pHUSH-Braf-shRNA [26] was re-engineered to express a Luc-BRAF transcript fusion. Dox-regulated luciferase expression was observed in several LOX-IMV1 pHUSH lines as indicated by the depletion of Luc-B-Raf fusion *in vitro *by a luciferase assay (additional file 8). We then performed an *in vivo *tumor growth study by subcutaneously inoculating Scid-beige mice with 1 × 10^6 ^cells of LOX-IMV1 shBraf.pGL3-Braf clone A1. After tumors had reached 250 mM^3^, the animals were separated into the appropriate Dox treatment group, and tumor growth and luciferase expression were monitored. As shown in additional file 9, control animals treated with sucrose water rapidly formed tumors, while those dosed with 500 μg/ml Dox had complete tumor remission according to caliper measurements of tumor volume. We have previously demonstrated that the in vivo tumor growth of LOX-IMV1 cells, engineered to express a luciferase targeting shRNA, are not affected by Dox treatment [26]. Therefore the observed tumor remission upon Dox treatment is due to Braf silencing and not the result of an off-target effect of Dox treatment. Animals treated with 20 μg/ml Dox exhibited growth stasis, while those treated with 10 μg/ml Dox resulted in an approximately 20% decrease in tumor growth relative to sucrose control animals. By measuring luciferase expression, a robust decrease in signal was measured at 500 μg/ml (98%) and a moderate decrease at 20 μg/ml Dox (62%) in comparison to the control or 10 ng/uL Dox treated animals (Figure 6D). Tumors were harvested at day 25 and *in vivo *Luc-Braf knockdown was confirmed in harvested tumor lysates by a luciferase assay, confirming the utility of this strategy to monitor as well as titrate target gene knockdown *in vivo *(Figure 6E).

## Discussion

We report the development of an inducible shRNA system that is delivered on a single plasmid or viral vector. By utilizing a reporter such as the pGL3-MELK construct, numerous shRNAs designed against a particular gene or genes can be screened, and effective hairpins can be rapidly cloned by Gateway^® ^recombination into the appropriate pHUSH destination vector. "First generation" pol III-shRNA vectors, "second generation" pol II-miRNA, and protein expression vectors have been successfully developed as components for inducible expression on several backbones. Silencing is robust and titratable for both *in vitro *and *in vivo *experimental models.

The described pHUSH system is based on the native Tet repressor instead of the TetR-VP16 fusion protein. Because regulation is based on repression of a modified active promoter rather than the transactivation of a minimal promoter, the required components are reliably expressed as a single construct. By combining the shRNA and TetR expression cassettes on the same plasmid or virus, we have dramatically decreased the time required to generate stable cell pools or clones exhibiting the desired level of regulated RNAi. Since we have not directly compared the pHUSH vector with other inducible shRNA, miRNA or protein expression systems, we cannot conclude if our vector design represents a major improvement over existing systems. However, by directly comparing the expression strategies of individual pHUSH vector components by a variety of cell-based assays, we can conclude that a single-plasmid vector system that combines miRNA expression, multiple TetR operons and a codon-optimized TetR is highly reliable for routine cell line engineering.

The ability to perform *in vitro *or *in vivo *RNAi high-throughput screens (HTS) may also be possible with the pHUSH system. This approach is based on the delivery of a large pool of shRNA or miRNAs designed against thousands of genes [5,39,40]. Several large, vector-based RNAi libraries now exist. Two are based on the first generation polIII-shRNA design [41,42], and one is based on a miR scaffold [18]. Recently a conditional shRNA library targeting approximately 2,500 genes and utilizing a two vector approach for inducible shRNA screens has been successfully employed [14]. It is therefore conceivable that that an effective single vector shRNA or miRNA conditional library could be based on the pHUSH system. Moreover, this combined system could also be used for direct *in vivo *application of inducible RNAi vectors for gene therapy studies or for inter-tumoral target validation in cancer mouse models.

By combining inducible shRNA technology with bioluminescence imaging, large *in vivo *experiments can be performed without the use of traditional caliper measurements [43]. We have previously demonstrated the utility of our approach by measuring the effect of oncogenic B-Raf depletion in a mouse model of metastatic melanoma. Pulmonary tumor burden was quantified by *in vivo *luciferase expression without animal dissection, thereby enabling measurements of disease progression over time in the same animal [26]. For the study presented in this report, endogenous B-Raf depletion was tracked by engineering a luceriferase-based reporter in which B-raf was cloned downstream of the luciferase stop codon. We demonstrate robust *in vivo *B-raf knockdown as detected by the decrease in luciferase signal while maintaining the reported tumor regression phenotype for endogenous B-Raf knockdown in this model.

One of the primary challenges to targeted cancer therapeutics is tumor escape mediated by drug resistance as in the case of Gleevec [44]. Inducible and reversible shRNA expression systems such as pHUSH may be utilized for tumor outgrowth or "Dox cycling" experiments in which tumors regress upon shRNA-mediated oncogene knockdown, acquire the ability to evade oncogene knockdown, and then progress as a resistant tumor [45]. The exogenous luciferase reporter allows the integrity of regulated gene silencing to be checked, ensuring that knockdown is maintained and that tumor escape is not simply caused by the loss of regulated shRNA expression. This approach, therefore, may be suited for tumor studies to model novel mechanisms of cancer resistance as well as identify new pathways for tumorigenesis. Importantly, our approach is also effective in a mouse model for brain cancer.

Several important *in vivo *applications for a single plasmid or viral vector system are not presented in this report. The question remains if the system would function as a germline-integrated cassette in adult mice. By engineering a second Tet operon in the H1 promoter, we have achieved regulated shRNA expression in embryoid bodies. The 2×-TetO_2 _H1 promoter exhibited total regulation of shRNA expression under differentiated conditions and decreased TetR levels. Although we have carefully optimized TetR translation by codon-optimization and TetR expression by selecting a promoter associated with strong and ubiquitous expression in multiple tissue and cell types, it is unclear whether the current vector configuration will yield transgenic mice with regulated shRNA expression in all tissues. However, the potential for the direct, *in vivo *application of a single viral vector system for inducible shRNA expression exists due to the co-integration of both the shRNA and TetR cassettes.

## Conclusion

The flexibility of the pHUSH to inducibly express shRNA, miRNA and proteins on a variety of plasmid and viral backbones will enable rapid target validation in a diverse array of *in vitro *and *in vivo *systems.

## Methods

Cell Lines and antibodies. HCT116, SVT2, LOX-IMV1, and A375 cells lines were maintained as previously reported [25,26]. 293T, 293HEK and NIH3T3 cells were obtained from American Type Culture Collection (Manassas, VA) and maintained at 37°C and 5% CO2 in DMEM with 10% tetracycline-free fetal bovine serum (Clontech, Palo Alto, CA) supplemented with 2 mM L-glutamine, 100 mM Hepes, and 100 U/ml penicillin/streptomycin. Anti-γ-tubulin, anti-BRAF, anti-phospho-Erk1/2, and anti-Erk2 antibodies were used for western blotting as previously described [26]. Monoclonal mouse IgG1 mix anti-TetR antibody was purchased from MoBiTec (Göttingen, Germany).

Generation of transgenic ES cell lines and ES cell in vitro differentiation. ES cell culture medium was made of Dulbecco's modified Eagle medium (DMEM), supplemented with 15% heat-inactivated fetal calf serum, 2 mM L-glutamine, 0.1 mM non-essential amino acids, 1000 IU/ml recombinant human leukemia inhibitory factor (LIF; ESGRO), 0.1 mM b-mercaptoethanol, 50 U/ml penicillin, and 50 ug/ml streptomycin. To generate transgenic ES cell lines, 1 × 10^7 ^of R1 ES cells were electroporated with 20 μg of linearized vector DNA and selected in ES cell culture medium containing 1–1.5 μg/ml puromycin. ES cell differentiation medium was made of Dulbecco's modified Eagle medium (DMEM), supplemented with 10% heat-inactivated fetal calf serum, 50 U/ml penicillin, and 50 ug/ml streptomycin. For in vitro differentiation, ES cells were first cultured in ES cell medium in hanging drops (600 cells/30 μl/drop) for one day and then in differentiation medium in suspension as embryoid bodies in bacteriological petri dishes for 7 days. Embryoid bodies were then transferred to tissue culture plates coated with 0.1% gelatin for continuous culture in differentiation medium. Colonies with beating cardiomyocytes were visually scored at 14–17 days post-induction of EB differentiation.

Vector Construction. The TetR gene was codon-optimized for mammalian expression using the DNAWorks program [46]. Using a compatible end derived from a *Bsa*I site, we ligated either the codon-optimized TetR or a PCR product encoding the original open reading frame of pcDNA6/TR (Invitrogen, Carlsbad, CA) in-frame to the *Nco*I start site of pDRIVE-5'RU (Invivogen, San Diego, CA). The human β-actin TetR cassette was then PCR amplified using Pfu Turbo Polymerase (Stratagene, La Jolla, CA), adding a four restriction site 'polylinker' (*Mfe*I, *Cla*I, *Spe*I, *Age*I) upstream of the promoter, and was subcloned into pIRES-Puro2 (Clontech, Palo Alto, CA) using *Mfe*I and *Eco*RI sites to form EV-pHUSH. Due to the possibility that 5' UTR of the human β-actin cassette harbored a splicing donor that may form an intron with the intervening sequence (IVS) [47] derived from pIRES-Puro2, we removed the IVS sequence by swapping the original IVS-IRES-Puro module with a related IRES-Puro fragment derived from pQCXIP (Clontech, Palo Alto CA).

shRNA hairpin shuttle vectors were constructed by PCR sub-cloning the pSuperior-H1 promoter (OligoEngine, Seattle, WA), adding *Xba*I, *Spe*I, and *Age*I sites, and then TOPO-cloning into pENTR/D (Invitrogen) to form pShuttle-H1-shRNA. In order to introduce hairpin sequences, pShuttle-H1-shRNA was linearized by cutting with *Bgl*II and *Hind*III, and hairpin inserts were generated by synthesizing complementary 5'-phosphorylated DNA oligonucleotides, which were annealed in 100 mmol/L potassium acetate, 30 mmol/L HEPES-KOH (pH 7.4), and 2 mmol/L magnesium acetate for 3 minutes at 95°C followed by 1 hour at 37°C. The resulting dsDNA fragment was ligated into pShuttle-H1-shRNA and confirmed by sequencing. A similar strategy was employed to add a second TetO_2 _operon to form pShuttle-H1-shRNA-2×-TetO_2 _by ligating dsDNA into the *Msl*I and *Hind*III sites of the orginal vector. H1-shRNA cassettes were subcloned into the pHUSH backbone by ligating the *Xba*I-*Age*I fragment from pShuttle-H1-shRNA into the compatible *Spe*I-*Age*I site of the pHUSH backbone to form the complete non-viral pHUSH vectors as presented in Figures 2 and 3.

The protein expression cassette was generated by PCR amplifying pcDNA4/TO, and the resulting product was TOPO-cloned into pENTR/D to form pShuttle-CMV/TO. To generate the inducible protein expression cassettes presented in Figure 4, an *Age*I (blunted with T4 polymerase)-*Eco*RI fragment from EGFP-C1 (Clontech, Palo Alto CA) was ligated to pShuttle-CMV/TO, which was prepared by cutting with *Pme*I and *Eco*RI to form pShuttle-CMV/TO-EGFP. To generate pShuttle-CMV/TO-EGFP-MELK^D150A^, the *Nhe*I-*Xba*I fragment comprising EGFP-MELK^D150A ^was cloned into the *XbaI *site of pShuttle-CMV/TO.

The inducible CMV-miR cassette was constructed by separately PCR amplifying pShuttle-CMV/TO and pmi6/EmGFP-miR-neg (Invitrogen, Carlsbad CA). Since these PCR products were designed to overlap each other, they were pooled and subjected to a second round of PCR, which resulted in ligation of the two products. The resulting PCR product was TOPO-cloned into pENTR/D (Invitrogen, Carlsbad CA) to form pShuttle-CMV/TO-miR. The original miRNA was replaced by ligating dsDNA oligos designed to restore the original sequence and maintain the appropriate overhangs for the introduction of new miRNA sequences. miRNAs were introduced by linearizing the vector with *Bsa*I.

To prepare the completed pHUSH retroviral vector utilized in Figures 4, 5, 6, 7, the *Bgl*II-*Eco*RI fragment of EV-pHUSH was subcloned into the retroviral vector pQCXIP, replacing the CMV promoter. The Gateway^® ^acceptor reading frame cassette B (Invitrogen, Carlsbad CA) was ligated to the *Mfe*I site, which was blunted by T4 polymerase, directly upstream of the human β-actin promoter to generate the final, Gateway^®^-compatible retroviral pHUSH system. Gateway^® ^cloning and plasmid propagation was preformed according to the manufacturer's recommendations.

The pGL3-Luciferase reporter vectors were generated by first ligating to the XbaI site of pGL3-Luc (Promega, Madison, WI) a mutiple cloing site downstream of the luciferase stop codon to form pGL3-Luc-MCS. pGL3-MELK, pGL3-BRAF or pGL3-p53 vectors were constructed by ligating the entire cDNA of MELK or BRAF into pGL3-Luc-MCS, or, in the case of pGL3-Luc-p53, long synthesized dsDNA. All oligonucleotide sequences employed in the generation of these vectors are available upon request.

H1-shRNA and CMV-miR hairpin selection and sequences. H1-shRNA hairpin sequences were either identified in previous reports, selected by standard 'Tuschl' rules [1], or custom designed by Dharmacon (Lafayette, CO). 19 bp core targeting sequences were converted to clonable dsDNA sequences with the aid of the program tohairpin (Colin Wanatabe, Genentech) and ordered as DNA oligonuceoltides. CMV-miR hairpins were designed using the BLOCK-iT RNAi Designer (Invitrogen, Carlsbad CA). Hairpin sequences for MELK, BRAF and luciferase were previously described [25,26]. For Melk knock-down in murine ES cells, (Figure 3B and Supplement Figure S3), the following DNA oligonucleotides were used: shMELK-Q1 (sense) 5'-GAT CCC CCG TGG ACT TCG TAC AGA AAT TCA AGA GAT TTC TGT ACG AAG TCC ACG TTT TTT GGA AA-3' and (antisense) 5'-GC TTT TCC AAA AAA CGT GGA CTT CGT ACA GAA ATC TCT TGA ATT TCT GTA CGA AGT CCA CGG GG-3'. Annotated shRNA and miRNA sequences used in Figures 1 and 4 are described in additional files 1 and 2.

Viral packaging and cell line generation. Retroviral packaging and transduction of target cell lines were prepared as described previously [25,26]. Briefly, to package retroviral stocks of pHUSH, AmphoPak cells (Orbigen, San Diego, CA) were transfected with 8 μg DNA using the CalPhos Mammalian Transfection kit (Clontech, Palo Alto CA). Supernatants were collected 48 h post-transfection, filtered through a 0.45 μm cellulose acetate filter and added to the target cell lines. In some cases, target cells were serially infected at 24 h intervals. Selection with puromycin began 48 h after the final infection and continued 5–7 days or until the cells were fully selected. After arriving at a stable pool, the cells were cloned by limiting dilution and assessed by qRT-PCR for regulated knockdown of the target gene.

Luciferase Assays. Transfection assays with luciferase assay endpoints were performed in duplicate or triplicate in 96-well plates at the plasmid molar ratios as listed in the results section. 293T or 293HEK cells were transfected using Lipofectamine 2000 with 200 ng of total DNA with 4 ng of the pRL plasmid, encoding the Renilla luciferase control, to normalize transfection efficiency between wells. The Steady-Glo Luciferase Assay System (Promega) was utilized with the Victor3 Multilabel Counter (Perkin-Elmer, Wellesley, MA) to quantify firefly and renilla luciferase expression 72 h post-transfection.

Knockdown and Phenotypic Analysis. RNA isolation and real-time qRT-PCR analysis was performed as previously described [25,26]. Additional Taqman primer and probe sequences used in report are murine p53 (forward primer, 5'-CCG CGG GCG TAA ACG-3', reverse primer, 5'-GCA TGG GCA TCC TTT AAC TCT AA-3', and probe 5'-FAM-CCT CAT TCA GCT CCC GGA ACA TCT C-BHQ-3') and murine SPF31 (forward primer, 5'-TGG GAA GCC ATC CTT GAG-3', reverse primer, 5'-TGA CTG CCG TGT GAA TCC-3', and probe 5'-FAM-CAA TAC CTC TCC TAT TGG AGC AAG GGC-BHQ-3'). Fluorescent detection of EGFP and EGFP-MELK^D150A ^proteins was conducted by fractionating equal total protein by polyacylamide gel electrophoresis on a 4–20% Tris-Glycine gel (Invitrogen, Carlsbad CA) and then imaged with the Typhoon Trio (GE Healthcare, Piscataway, NJ).

To determine whether attenuation of oncogenic BRAF by inducible-shRNA knockdown decreases A375 melanoma cell proliferation, A375 cells expressing either BRAF or control GFP shRNAs were cultured in 0.1% serum in the presence or absence of 0.5–1 μg/ml Doxycycline. At 2-day intervals, viable cell counts were determined by the tryphan blue exclusion method using a Vi-Cell Analyzer (Beckman Coulter). Cell numbers are presented as mean ± SD.

The concentration VEGF_165–206 _was analyzed in cell lysates homogenized in buffer containing 10 mM Tris, 0.1% monothioglycerol acetate, and 1.5 mM EDTA (pH 7.4). The homogenate was then centrifuged for 30 min at 200,000 × *g *at 4°C and VEGF measured in the resulting supernatant using quantitative ELISAs. The VEGF_165–206 _ELISA uses a mouse monoclonal antibody (3.5F8) that detects exon 7-encoded peptide sequences specific for VEGF_165 _for capture and biotinylated A4.6.1 for detection [48].

Tumor models. Six- to 8-week-old female SCID Beige or CD-1 Nude mice were purchased from Charles River Laboratories and maintained in accordance to Guidelines for the Care and Use of Laboratory Animals in our institute's conventional animal facility. For subcutaneous tumor models, mice were injected in the right flank with 3 × 10^6 ^human LOX-IMVI shRNA-containing cell clones resuspended into 200 μL phosphate-buffered saline (PBS). When tumors reached a mean volume of ~150 mm^3 ^the mice with similarly sized tumors were grouped into treatment cohorts. Mice received 5% sucrose only or 5% sucrose plus 0.01, 0.02 or 0.5 mg/ml doxycycline for control and knockdown cohorts, respectively. All water bottles were changed 3 times per week. Tumors were measured with calipers and mice weighed twice per week. Mice whose tumors reach 2000 mm^3 ^were euthanized. At the end of the dosing study, or as indicated, appropriate tumor samples were taken. Between 3–4 mice were used for each treatment group and results are presented as mean tumor volume ± SEM.

Intracranial implantation. Cells were harvested in mid-log phase and resuspended in Hank's buffer. CD-1 nude mice were anesthetized with 2% isoflurane and placed into the stereotactic apparatus before exposure of the cranium with a midline incision. A less than 1 mm burrhole was drilled through the parietal bone. 2.5 × 10^5 ^cells in 5 μl were injected in the right striatum at coordinates from the bregma 0.5 mm posterior, 2.0 mm lateral, and 3.5 mm intraparenchymal over 5 minutes using 10 ul Hamilton syringe with a 27 g stainless steel cannula. The incision was closed with suture.

### Bioluminescence imaging

Mice were imaged using the following procedure to monitor doxycycline mediated knockdown. Prior to image acquisition, tumor-bearing mice were anesthetized using isoflurane and injected intraperitoneally with 200 mg/kg D-luciferin (Invitrogen; Carlsbad, California) in 250 μL volume. During image acquisition the mice were placed in a light-tight imaging chamber and maintained on isoflurane anesthesia via nose cone delivery and body temperature was regulated using a warming pad. Bioluminescence images were acquired using a cooled intensified charge-coupled device camera. Image acquisition times varied according to signal intensity but were typically less than 1 second for the subcutaneous model with longer integration times used for the intracranial model. Signals were localized by overlaying a reference image of the mouse with the bioluminescence data image. Images were quantitated by evaluating pixel intensities in the bioluminescence data image, applying an appropriate background correction and scaling the resulting values to account for variations in acquisition time and camera settings to give a mean intensity in relative light units (RLU) that could be further analyzed to discern the effects of doxycycline mediated luciferase knockdown.

## Abbreviations

RNAi, RNA interference; 

siRNA, short-interfering RNA; 

shRNA, short-hairpin RNA; 

Pol III, RNA polymerase III; 

TetR, Tetracycline Repressoe; 

miRNA, micro-RNA; 

EmGFP, emerald GFP; 

DOX, doxycycline; 

BLI, bioluminescence imaging.

## Competing interests

The author(s) declares that there are no competing interests.

## Authors' contributions

DG and DD designed and engineered the pHUSH vectors. KH, ME, SS and LM ran in vitro and in vivo studies focusing on Braf knock-down. LP, NK, AG and MC developed methods for in vivo BLI. ZG performed ES cell experiments. All authors have read and approved the final manuscript.

## Supplementary Material

Additional file 1 Target and hairpin transcript sequence for Melk directed H1-shRNAs. Schematic comparison of shRNA formatting. Click here for file

Additional file 2 Target and hairpin transcript sequence for p53 directed H1-shRNAs versus CMV shRNAmirs. Schematic comparison of shRNA versus miRNA formatting. Click here for file

Additional file 3 Melk knockdown efficiency in ES cell clones. Comparison of doxycycline regulated Melk knockdown between 1× and 2× TetO2 modified H1 promoters. Click here for file

Additional file 4 Optimized selection improves H1-shRNA mediated knockdown. Comparison of average Braf knockdown efficiency in cells selected at 2 versus 5 ug/ml Puromycin. Click here for file

Additional file 5 Dose dependent titration of H1-shRNA silencing in vitro. Titration of doxycycline mediated silencing of luciferase expression. Click here for file

Additional file 6 pHUSH: a single vector system for conditional gene expression. Schematic representation of modular features comprising the completed pHUSH. Click here for file

Additional file 7 Dose dependent titration of H1-shRNA silencing in vivo. Titration of doxycycline mediated silencing of luciferase expression within subcutaneous and intracranial tumor models. Click here for file

Additional file 8 Generation of a luciferase reporter cell line to monitor doxycycline regulated shRNA expression. Comparison of doxycycline regulated expression of a luciferase-Braf transcript fusion in shCom-4-pHUSH LOX-IMV1 clones. Click here for file

Additional file 9 In vivo tumor growth of shCom-4-pHUSH LOX-IMV1 cells engineered with the luciferase-Braf shRNA reporter. Correlation between calliper and BLI measurements validate the utility of the luciferase reporter as a method for quantifying in vivo tumor growth. Click here for file
