# Supplementary figures and images for: pHUSH: a single vector system for conditional gene expression
Source: BMC Biotechnol. 2007 Sep 26;7:61. doi: 10.1186/1472-6750-7-61 (PMC2174931; doi:10.1186/1472-6750-7-61)

## Slide 1
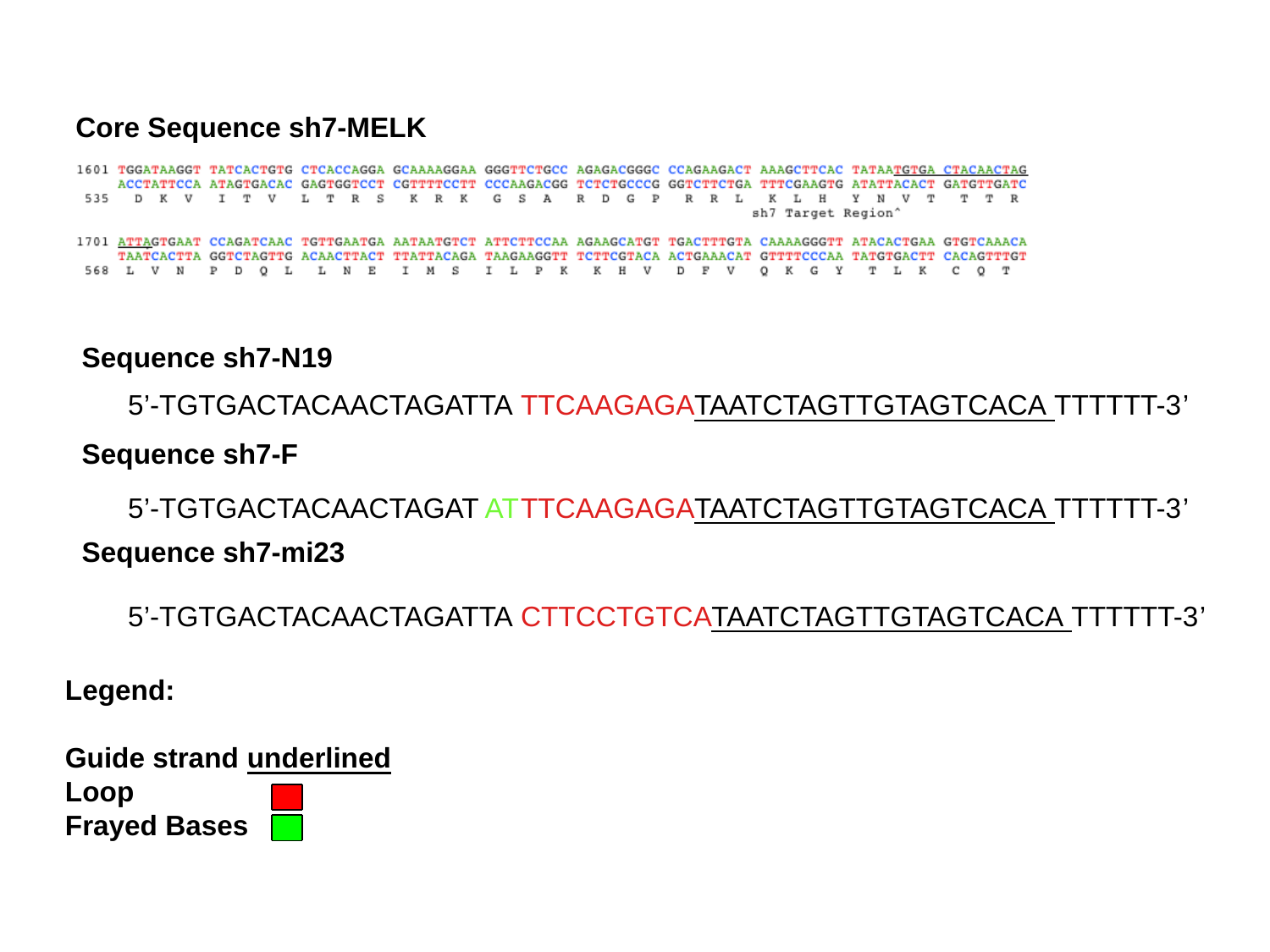

## Slide 2
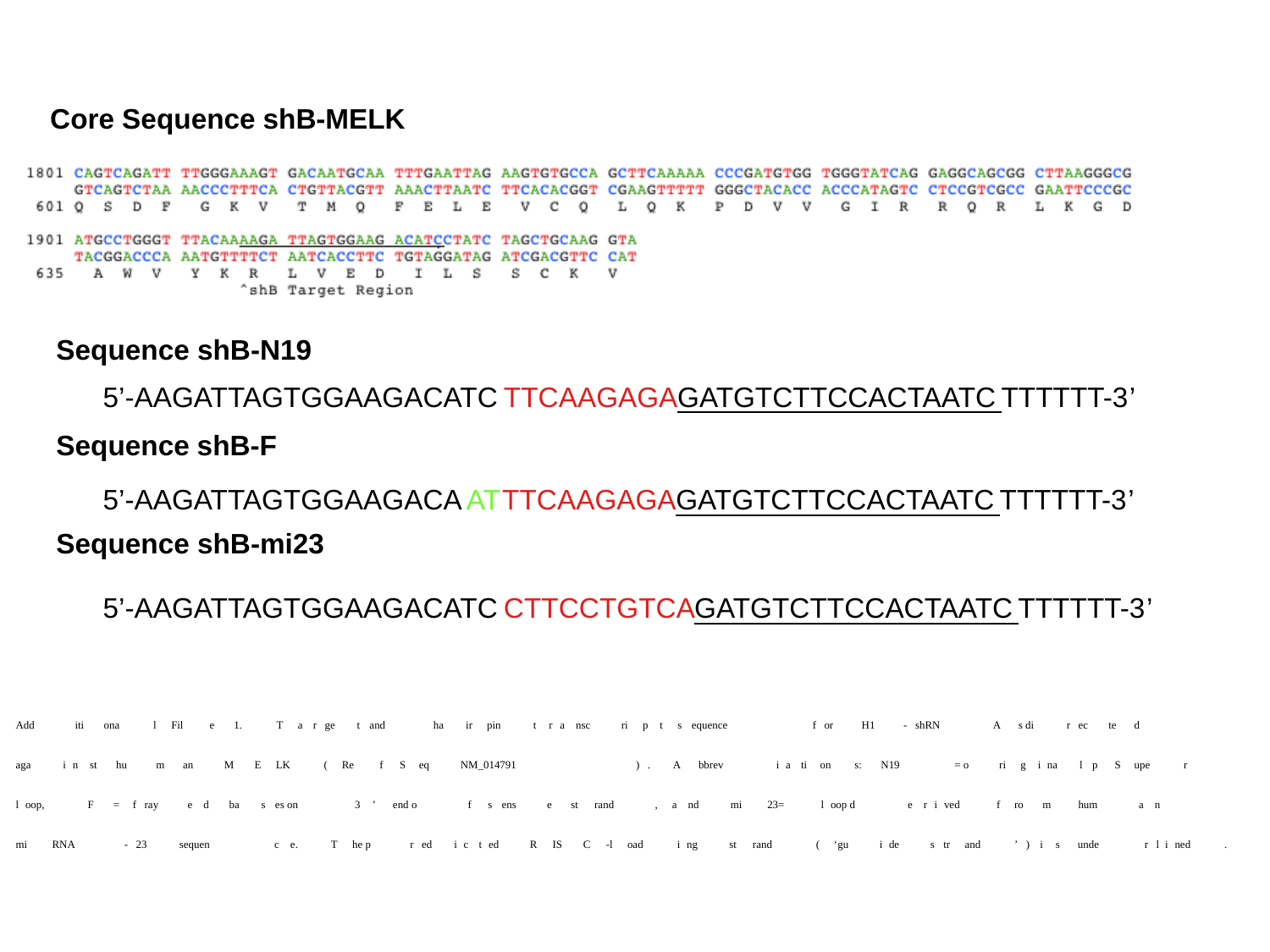

Supplement: Additional file 1 — Target and hairpin transcript sequence for Melk directed H1-shRNAs. Schematic comparison of shRNA formatting. [file 1472-6750-7-61-S1.ppt]

## Slide 1
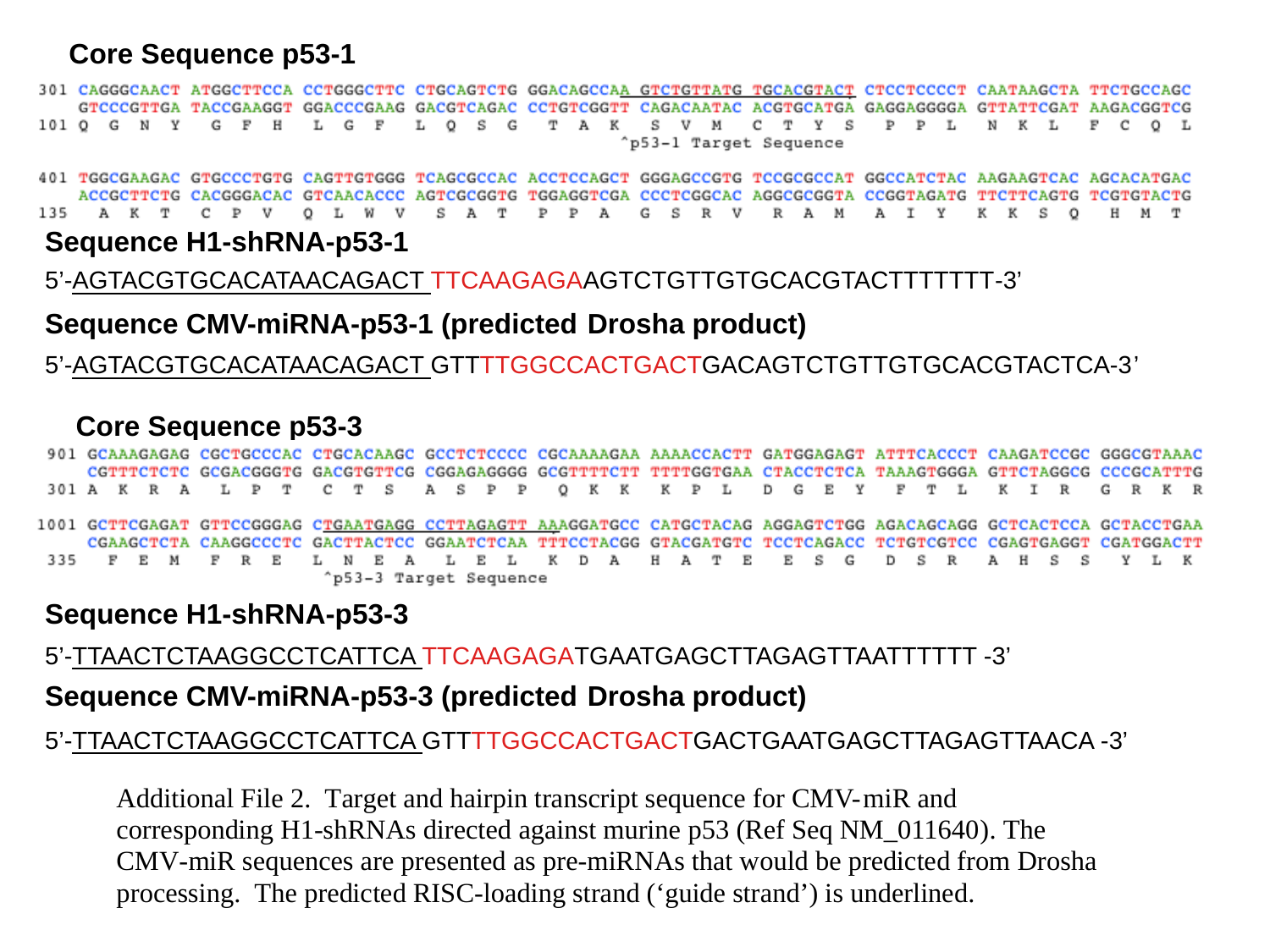

Supplement: Additional file 2 — Target and hairpin transcript sequence for p53 directed H1-shRNAs versus CMV shRNAmirs. Schematic comparison of shRNA versus miRNA formatting. [file 1472-6750-7-61-S2.ppt]

## Slide 1
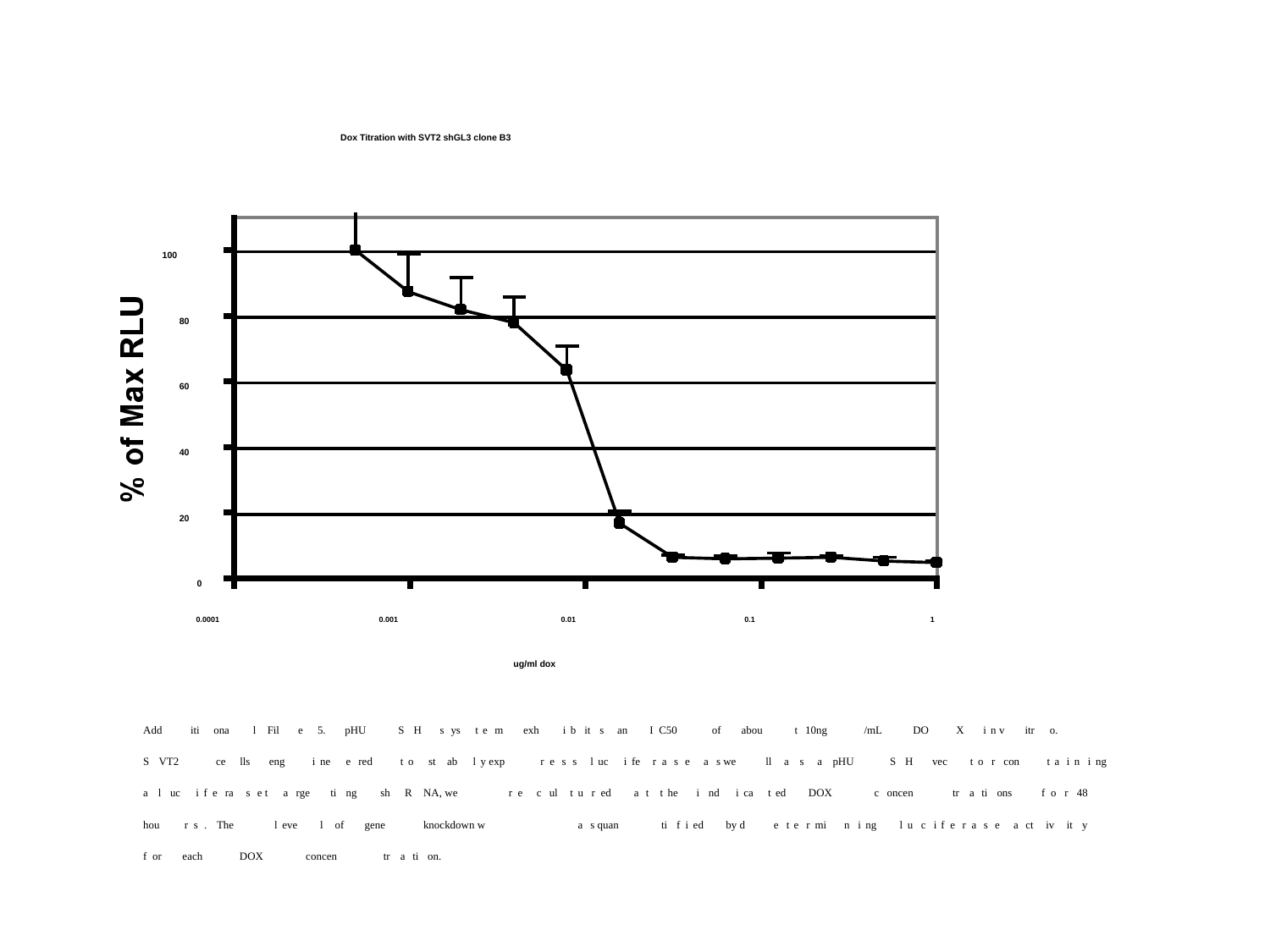

Supplement: Additional file 5 — Dose dependent titration of H1-shRNA silencing in vitro. Titration of doxycycline mediated silencing of luciferase expression. [file 1472-6750-7-61-S5.ppt]

## Slide 1
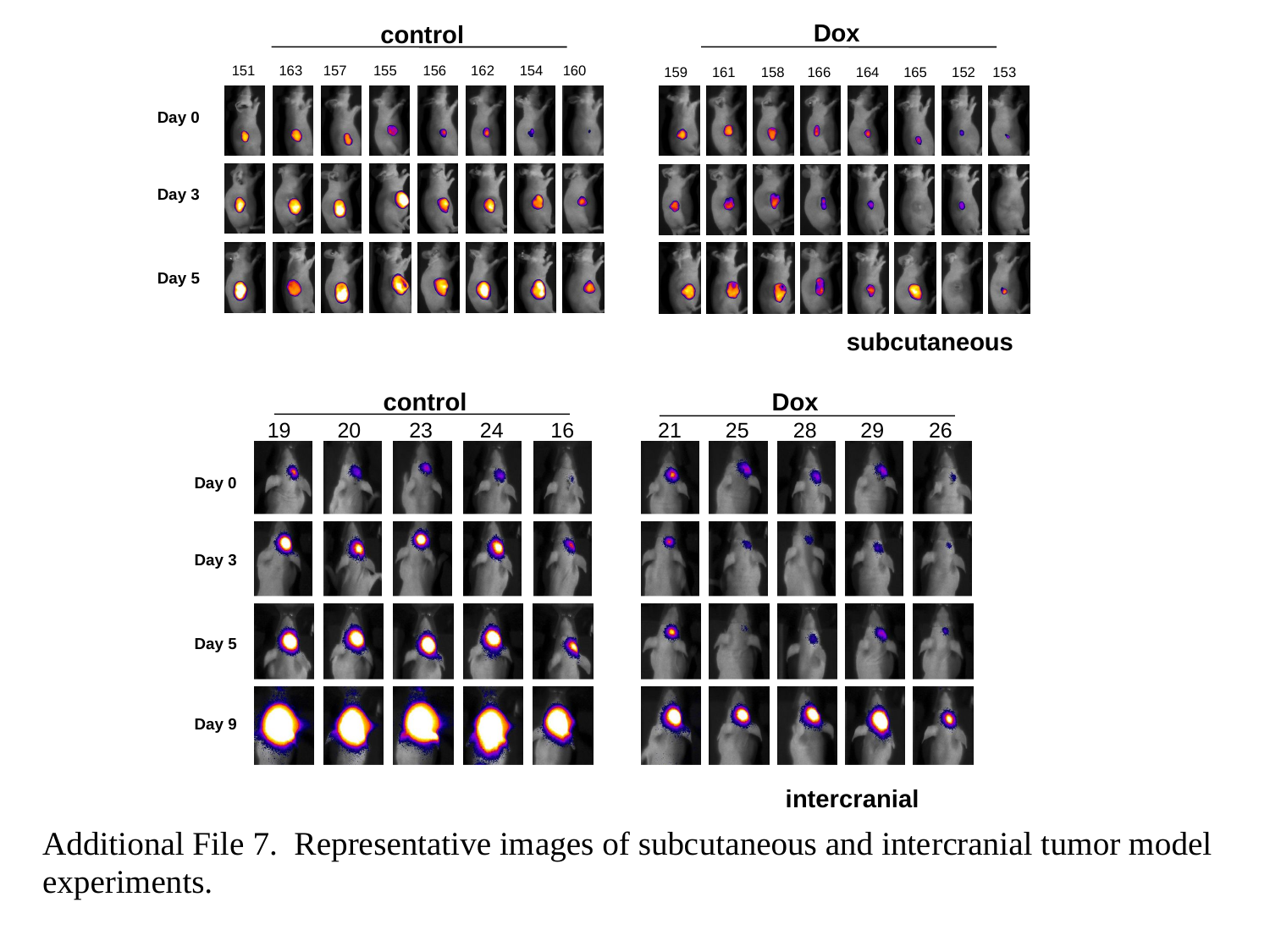

Dox
control
151
163
157
155
156
162
154
160
159
161
158
166
164
165
152
153
Day 0
Day 3
Day 5
subcutaneous
control
Dox
19
20
23
24
16
21
25
28
29
26
Day 0
Day 3
Day 5
Day 9
intercranial

Supplement: Additional file 7 — Dose dependent titration of H1-shRNA silencing in vivo. Titration of doxycycline mediated silencing of luciferase expression within subcutaneous and intracranial tumor models. [file 1472-6750-7-61-S7.ppt]

## Slide 1
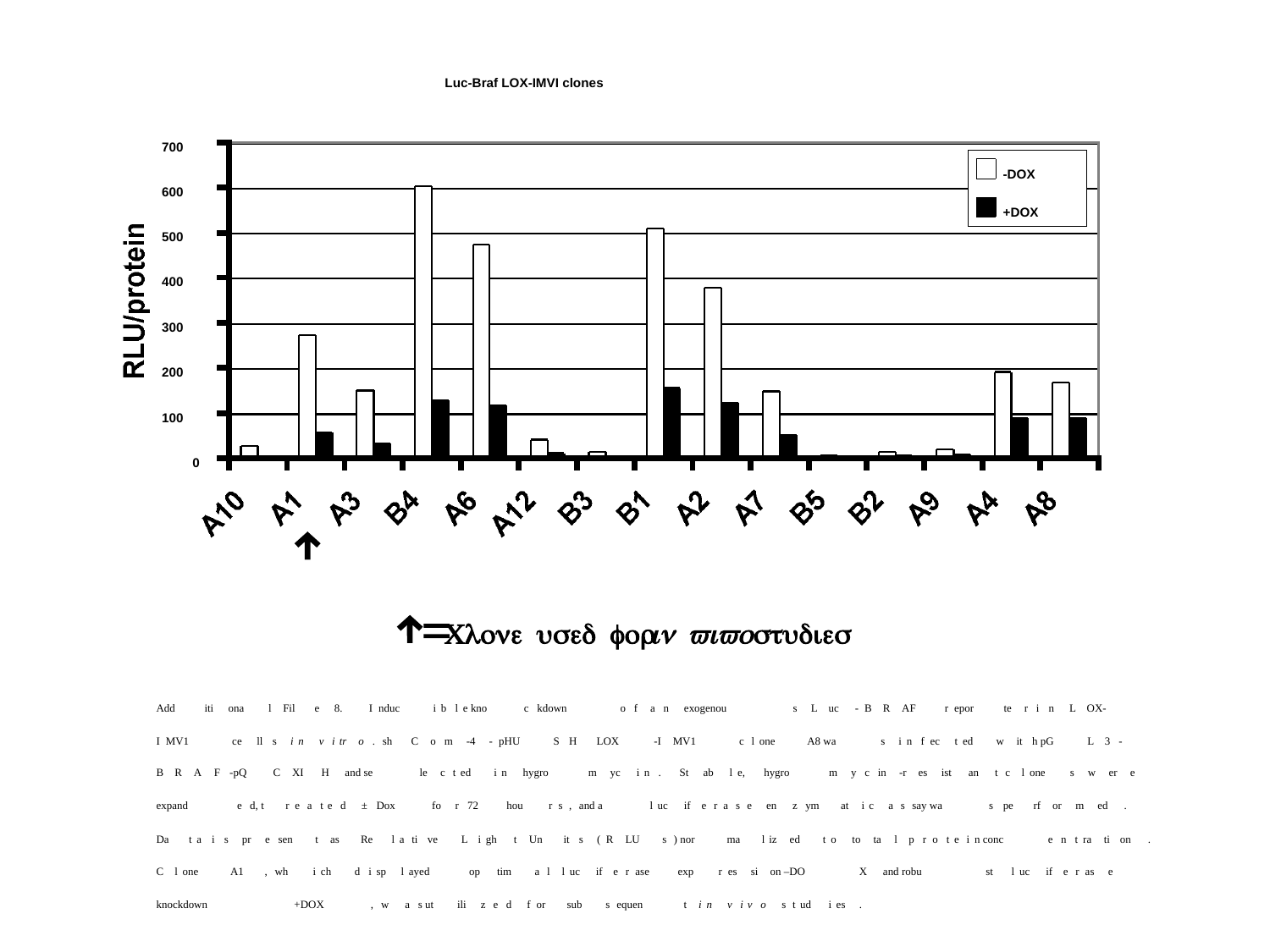

Supplement: Additional file 8 — Generation of a luciferase reporter cell line to monitor doxycycline regulated shRNA expression. Comparison of doxycycline regulated expression of a luciferase-Braf transcript fusion in shCom-4-pHUSH LOX-IMV1 clones. [file 1472-6750-7-61-S8.ppt]

## Slide 1
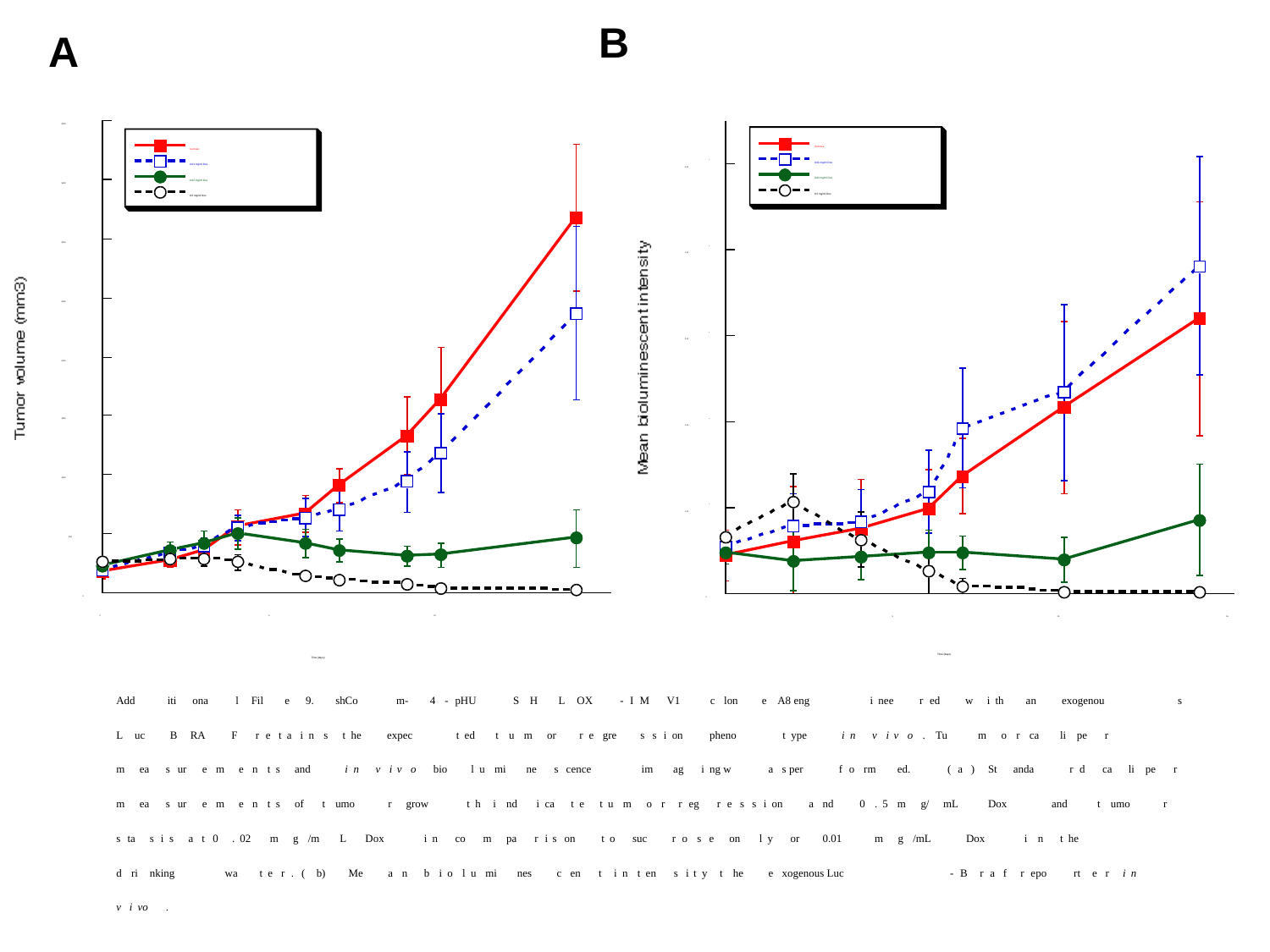

Supplement: Additional file 9 — In vivo tumor growth of shCom-4-pHUSH LOX-IMV1 cells engineered with the luciferase-Braf shRNA reporter. Correlation between calliper and BLI measurements validate the utility of the luciferase reporter as a method for quantifying in vivo tumor growth. [file 1472-6750-7-61-S9.ppt]
