# Supplementary material for: pHUSH: a single vector system for conditional gene expression
Source: BMC Biotechnol. 2007 Sep 26;7:61. doi: 10.1186/1472-6750-7-61 (PMC2174931; doi:10.1186/1472-6750-7-61)
Supplement: Additional file 3 — Melk knockdown efficiency in ES cell clones. Comparison of doxycycline regulated Melk knockdown between 1× and 2× TetO2 modified H1 promoters. [file 1472-6750-7-61-S3.pdf]

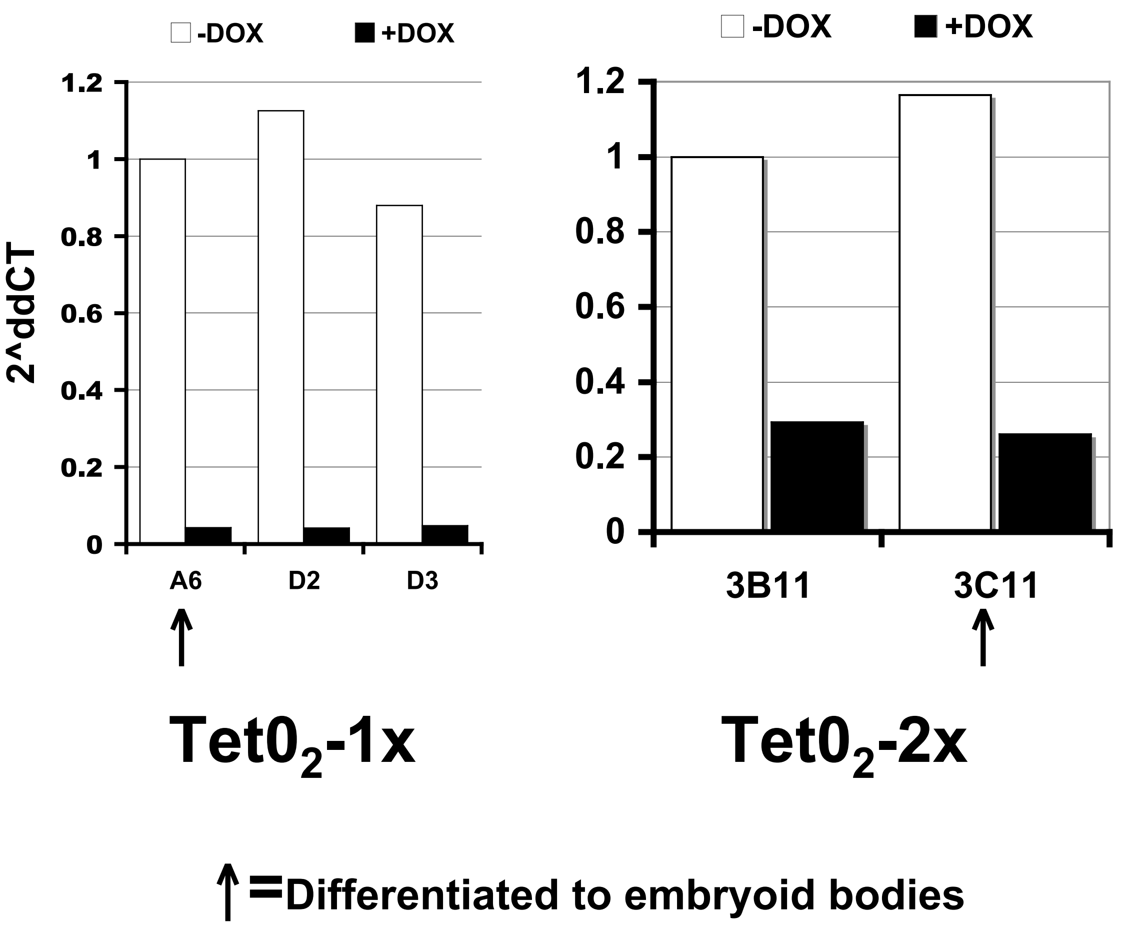

Additional Figure 3. Robust knockdown in undifferentiated murine embryonic stable clones derived with 1x and 2x-TetO_2 pHUSH vectors. Briefly, ES cells were electroporated and selected in 1-1.5ug/mL puromycin as described in the Methods section. Clones were first selected on the basis on high TetR expression, expanded and treated ±Dox for 48 hours. Knockdown was determined by qRT-PCR.
