# Supplementary material for: pHUSH: a single vector system for conditional gene expression
Source: BMC Biotechnol. 2007 Sep 26;7:61. doi: 10.1186/1472-6750-7-61 (PMC2174931; doi:10.1186/1472-6750-7-61)
Supplement: Additional file 4 — Optimized selection improves H1-shRNA mediated knockdown. Comparison of average Braf knockdown efficiency in cells selected at 2 versus 5 ug/ml Puromycin. [file 1472-6750-7-61-S4.pdf]

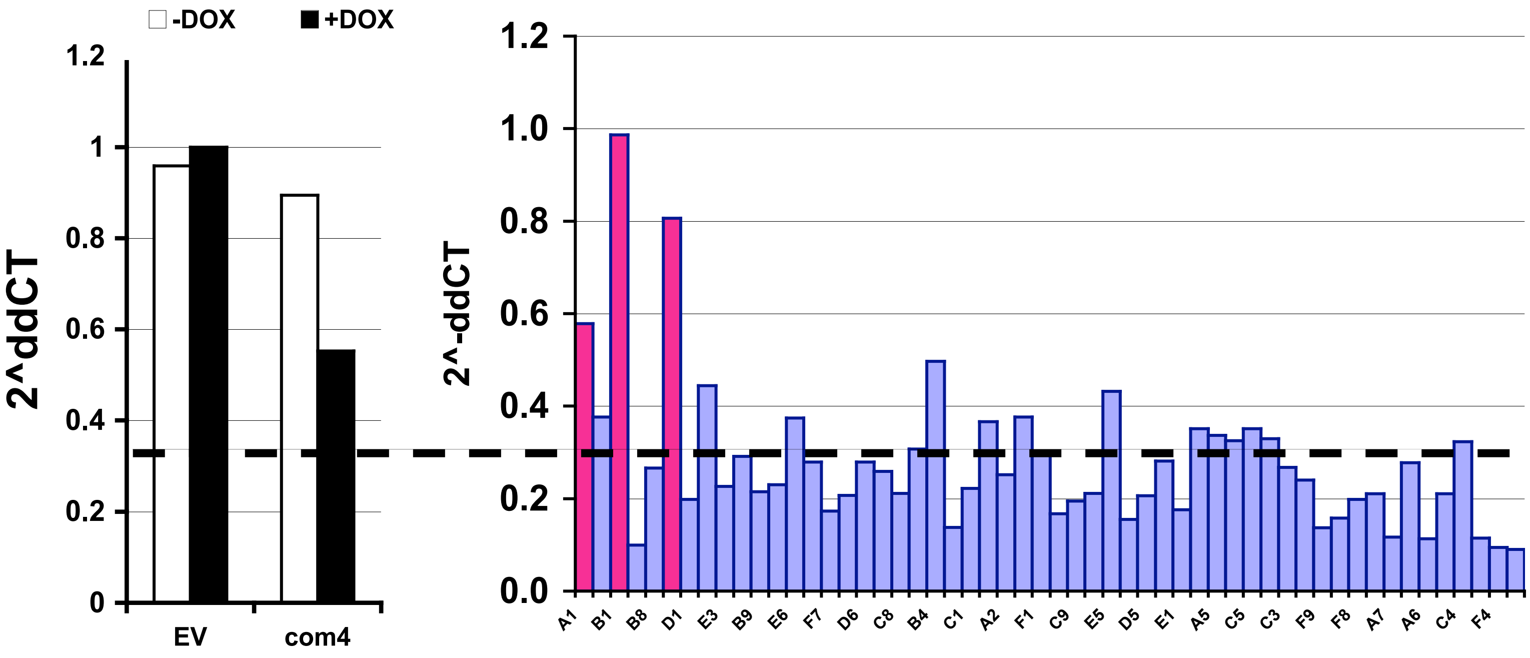

Selection at 2ug/mL (43% knockdown) **at 5ug/mL puromycin (73% average knockdown)**

Addition File 4. Optimized selection pressures improves H1-shRNA mediated knockdown in LOX-IMV1 melanoma cells. By enhancing selection pressure with increasing amounts of puromycin (2ug/mL to 5ug/mL), robust B-Raf knockdown was observed by qRT-PCR. Stables pools at 2ug/mL puromycin were compared to the average knockdown of 54 clones derived from a pool selected at 5ug/mL puromycin. Cells were treated ±Dox for 72 hours. Red bars indicate only three clones with less than 50% knockdown upon DOX addition.
