# Supplementary material for: pHUSH: a single vector system for conditional gene expression
Source: BMC Biotechnol. 2007 Sep 26;7:61. doi: 10.1186/1472-6750-7-61 (PMC2174931; doi:10.1186/1472-6750-7-61)
Supplement: Additional file 6 — pHUSH: a single vector system for conditional gene expression. Schematic representation of modular features comprising the completed pHUSH. [file 1472-6750-7-61-S6.pdf]

Retroviral system

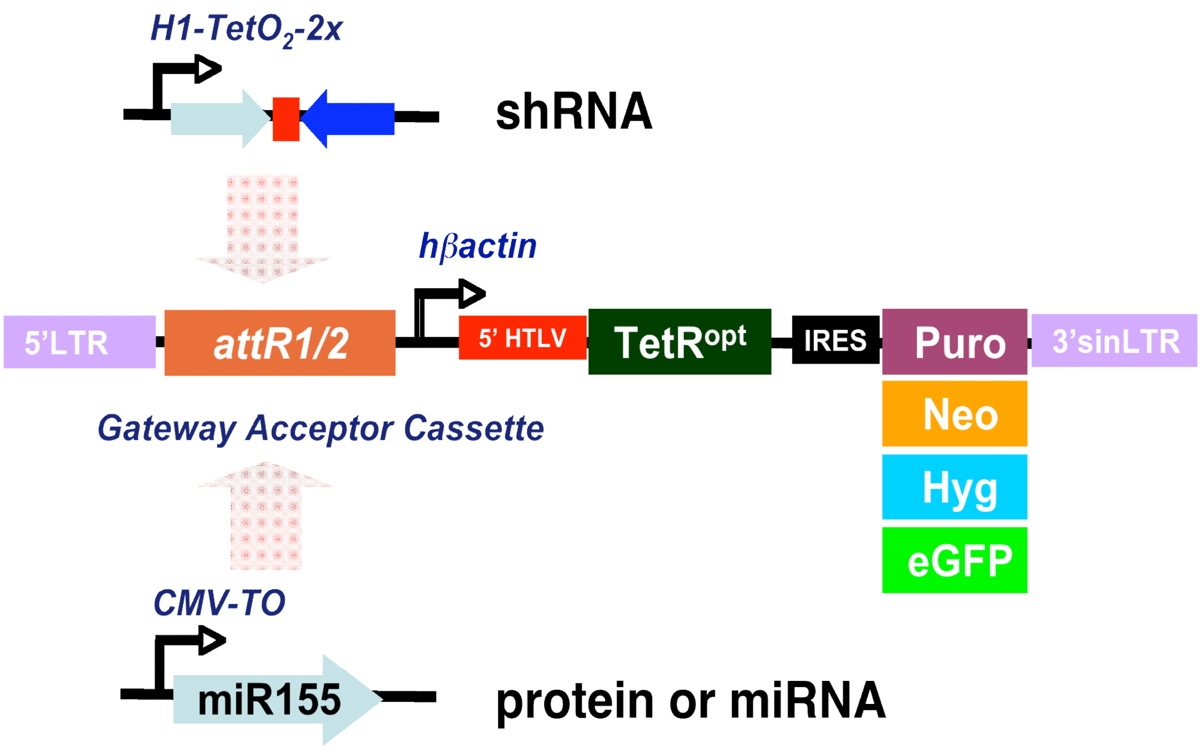

Additional File 6. Complete pHUSH vector system for the inducible expression of shRNA, miRNA, and proteins on a retroviral backbones.
